# Disseminated *Nocardia Paucivorans* in an immunocompetent patient: A case report and literature review

**DOI:** 10.1002/ccr3.4659

**Published:** 2021-08-06

**Authors:** Mohammed Samannodi

**Affiliations:** ^1^ Department of Medicine College of Medicine Umm AlQura University Makkah Saudi Arabia

**Keywords:** abscess, mass, Nocardia, sequencing

## Abstract

*Nocardia paucivorans* can cause disseminated infection in immunocompetent hosts in rare occasions. *Nocardia paucivorans* is usually susceptible to many antibiotics including Trimethoprim/Sulfamethoxazole. Duration of treatment is usually 6–12 months.

## Graphical abstract

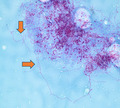

## 1 BACKGROUND

*Nocardia paucivorans* is uncommon found species and has been shown to cause localized and disseminated infections. We report a 73‐year‐old immunocompetent male patient who developed lung mass, two brain abscesses, and right knee abscess caused by *N. paucivorans*. Patient had favorable response to antimicrobial therapy and discharged home with outpatient follow‐up.

The majority of nocardia infections occur in immunocompromised hosts. However, up to one‐third of patients with nocardiosis are immunocompetent.^1^ *Nocardia paucivorans* is an uncommon species with the ability to cause severe infection in humans. It can cause different forms of infection, ranging from localized infection such as cutaneous or pulmonary to severe disseminated infection that usually involve central nervous system (CNS). In general, *N*. *paucivorans* is susceptible to the common antibiotics used to treat nocardiosis.^2^ To our knowledge, 40 cases have been reported in the literature, both in immunocompromised and in immunocompetent individuals.

## 2 CASE PRESENTATION

73‐year‐old male patient from California, USA, admitted to our hospital in Houston, Texas, USA, for right lung mass evaluation and mild intermittent headache. Two months ago, patient was treated for pneumonia with a short course of oral levofloxacin. Then, the symptoms recur in one week after finishing the antibiotic course. His past medical and past surgical history are unremarkable. Two months ago, he received a 5‐day course of oral levofloxacin and currently he denied taking any medications. Chest X‐ray and CT chest were performed by his primary care doctor and revealed right middle lobe mass. Six months ago, patient was in Alaska and Argentina for hunting ducks and fishing. Patient smokes 30 pack‐year. He denied drug abuse, sick contact, pets, or tick bites.

On physical examination, his blood pressure was 130/70 mmHg, his temperature was 99.2 F, his pulse was 80 beats per minute, and his respiratory rate was 16 breath per minute. Respiratory examination revealed decreased breath sounds on the right middle lobe. Rest of cardiovascular, neurological, abdominal, and skin examinations were unremarkable. At this point, neoplastic disease was on top of our differential diagnosis list followed by other diseases such as infections or autoimmune disorders.

**FIGURE 1:**
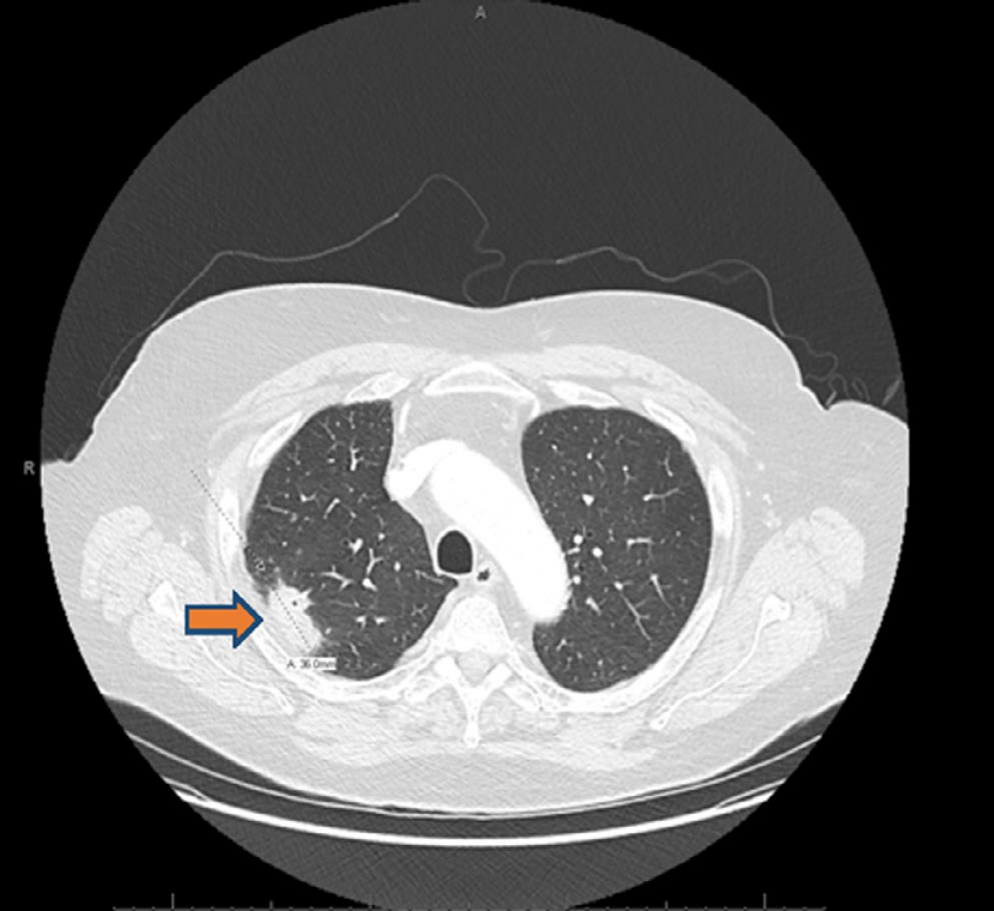
CT chest revealed 3.6 cm right middle lobe mass

**FIGURE 2:**
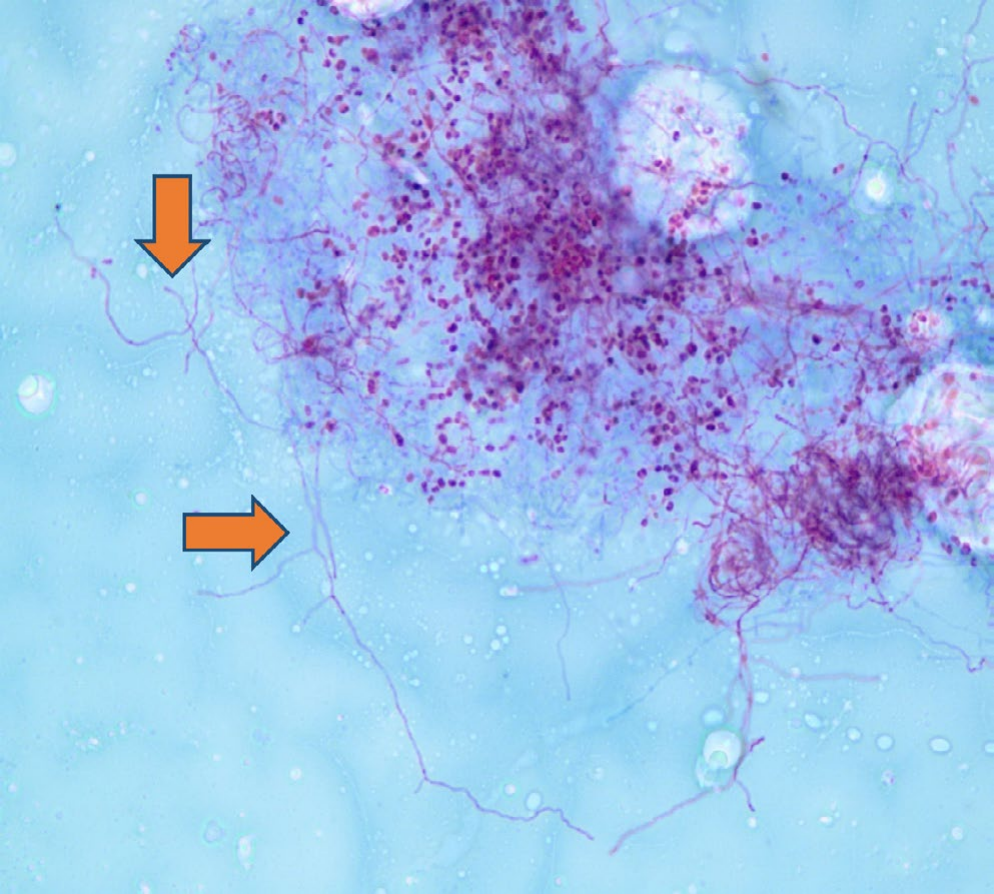
Gram staining of right middle lobe mass revealed branching gram‐positive rods consistent *Nocardia* species

**FIGURE 3:**
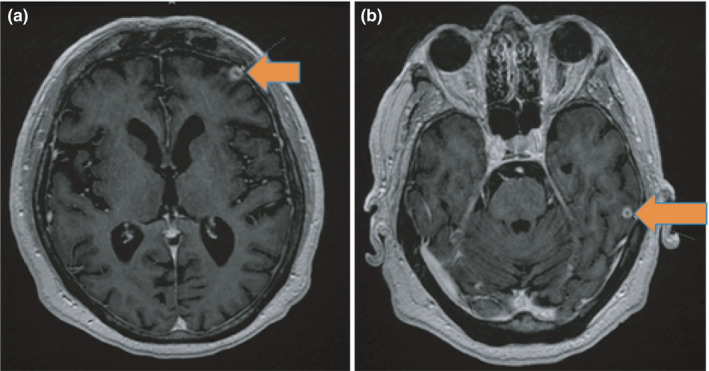
MRI of brain with contrast. (A) 0.8 cm ring‐enhancing lesion in left frontal cortex. (B) 0.6 cm ring‐enhancing lesion in left temporal lobe

**FIGURE 4:**
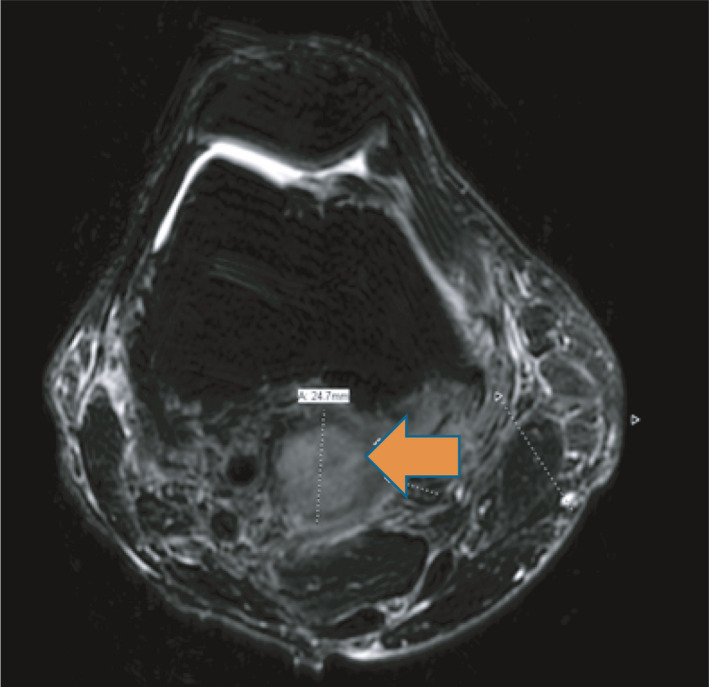
MRI of right knee revealed 2.5 X 2.4 cm ill‐defined mass involving the distal gastrocnemius muscle

Initial complete blood count and complete metabolic panel were normal. Serum interferon gamma release assay (IGRA), serum Beta‐d‐Glucan, serum aspergillus galactomannan antigen, serum coccidioides antibodies, and urine histoplasma antigen were negative. CT chest revealed 3.6 cm right middle lobe mass (Figure 1). Patient underwent excisional lung biopsy which showed branching gram‐positive rods consistent *Nocardia* species (Figure 2). We started intravenous imipenem and oral Trimethoprim/Sulfamethoxazole. CT head with contrast was unremarkable. For intermittent headache, MRI brain was performed and showed 0.8 cm ring‐enhancing lesion in left frontal cortex (Figure 3A) and 0.6 cm ring‐enhancing lesion in left temporal lobe (Figure 3B). In the sixth day of hospitalization, patient started to complain of posterior right knee pain and lump. MRI of right knee revealed 2.5 X 2.4 cm ill‐defined mass involving the distal gastrocnemius muscle (Figure 4). Right posterior knee mass fine needle aspiration was performed and resulted as fibroadipose tissue with granulation tissue, plasma cells, macrophages, and neutrophils. Culture of the aspirated fluid showed no growth. HIV test was negative, and CD4 count was normal.

Later, the culture of the lung mass grew *Nocardia paucivorans* by using 16S ribosomal sequencing and the susceptibility is reported in (Table 1). Patient was discharged on IV imipenem and TMP/SMX until resolution of the headache (8 weeks). Then, the treatment course was continued for at least one year with oral TMP/SMX alone with close monitoring to the major side effects such as agranulocytosis, thrombocytopeneia, electrolytes abnormalities, and renal impairment. Patient is following up with an infectious disease specialist at his home town. Patient reported complete resolution of his symptoms at the 8th week of treatment no side effects were reported.

**TABLE 1:** Minimal inhibitory concentrations of various antibiotics for *Nocardia Paucivorans*

Antibiotics	Minimal inhibitory concentrations (mcg/mL)	Interpretation
Amoxicillin‐clavulanate	16/8	Intermediate
Cefepime	4	Sensitive
Ceftriaxone	≤4	Sensitive
Imipenem	≤2	Sensitive
Ciprofloxacin	≤ 0.12	Sensitive
Moxifloxacin	≤ 0.25	Sensitive
Amikacin	≤ 1	Sensitive
Tobramycin	≤ 1	Sensitive
Doxycycline	0.25	Sensitive
Minocycline	≤ 1	Sensitive
Trimethoprim/Sulfamethoxazole	≤ 0.25/4.75	Sensitive
Linezolid	≤ 1	Sensitive
Clarithromycin	0.25	Sensitive

## 3 DISCUSSION

*Nocardia* species are branching, filamentous gram‐positive bacilli that grow aerobically. They can be found in soil, decomposing vegetation and decaying organic matter and water.^3^ *Nocardia* is an opportunistic pathogen, with majority of infections occurring in immunocompromised hosts. Patients with human immunodeficiency virus infection, solid‐organ or hematopoietic stem cell transplant or those receiving immunosuppressive medications are at high risk for nocardiosis.^4^, ^5^ However, up to one‐third of patients with nocardiosis are immunocompetent such as our case.^1^ Pulmonary nocardiosis is the most common clinical manifestation because inhalation is the primary route of infection. Disseminated nocardiosis is highly associated with immunocompromised conditions, and majority of cases in the literature were due to hematogenous spread. Nocardiosis in immunocompetent patients is usually localized, and dissemination is uncommon.^1^, ^2^

Our case presentation was an example of disseminated nocardiosis from a primary pulmonary infection and spread to brain and soft tissues of right knee. In 2014, Hommoud M. et al performed a detailed literature review on *N*. *paucivorans* reports. He found disseminated disease was reported in 11 cases. In cases of disseminated infections, CNS involvement occurred in 9 of 11.^6^ Our case is supporting the previous reports in which CNS involvement is common in infection by *N*. *paucivorans*.

Diagnosis of *N*. *paucivorans* in our patient was identified by using 16s ribosomal sequencing of lung mass tissue. Despite negative culture result of right knee mass aspiration, we still believe that right knee mass was caused by *N*. *paucivorans*. The negative culture result of right knee mass was likely because patient was on effective antibiotics for 6 days prior to aspiration which can potentially inhibit bacterial growth.

It is recommended by many experts to initiate treatment for CNS or disseminated nocardiosis with combination of two or three effective antibiotics until susceptibility results are available. In disseminated nocardiosis, it is advised to continue parenteral antibiotics therapy for three to six weeks and clinical improvement should be seen before changing to an oral regimen. Thereafter, maintenance therapy with one or two oral effective antibiotics can be continued for 6–12 months in immunocompetent patients. *Nocardia paucivorans* is usually susceptible to many antibiotics that have anti‐nocardiosis effect specifically TMP/SMX which is the main key of treatment.^2^, ^7^, ^8^, ^9^, ^10^ Our case demonstrated significant clinical improvement by the 8th week which we have discontinued IV imipenem and continued oral TMP/SMX alone for at least one year due to multifocal lesions with CNS involvement.

## 4 CONCLUSION

*Nocardia paucivorans* has the ability to cause disseminated infection in immunocompetent hosts.

## AUTHOR CONTRIBUTIONS

MS contributed to conception and design of the study, acquisition and analysis of data, literature review and drafting the whole manuscript.

## ETHICAL STATEMENT

I testify that my article submitted to Clinical Case Reports Journal.

1. This material has not been published in whole or in part elsewhere;
2. The manuscript is not currently being considered for publication in another journal;
3. I have been personally and actively involved in substantive work leading to the revised manuscript, and will hold themselves jointly and individually responsible for its content.

## Data Availability

The data that support the findings of this study are openly available at [DOI] and reference number [CCR3‐2021–06–0944].
